# Supplementary material for: Microbial communities respond to experimental warming, but site matters
Source: PeerJ. 2014 Apr 24;2:e358. doi: 10.7717/peerj.358 (PMC4006228; doi:10.7717/peerj.358)
Supplement: Supplemental Information 1 [file peerj-02-358-s001.pdf]

Appendix 1. Microbial community structure and function differed significantly between the northern and southern sites. F and p statistics (in parentheses) show the main effects of site, soil temperature, and soil moisture and the interactive effects of site × soil temperature, site × soil moisture, soil temperature × soil moisture, and site × soil temperature × soil moisture on all variables measured. P values ≤ 0.05 are bold. The PERMANOVA community analysis used site as a factor and soil temperature and soil moisture as covariates thus there are no F and p values for the full model.

**Full model**		**Site**	**Soil temperature**	**Soil moisture**	**Site × soil temperature**	**Site × soil moisture**	**Soil temperature × soil moisture**	**Site × soil temperature × soil moisture**
Microbial community composition	na	**1.67**<br>**(0.03)**	**17.86**<br>**(<0.01)**	0.87<br>(0.63)	0.81<br>(0.71)	0.82<br>(0.69)	1.03<br>(0.42)	0.82<br>(0.69)
Fungal community composition	na	0.85<br>(0.66)	**4.33**<br>**(<0.01)**	0.92<br>(0.58)	1.10<br>(0.35)	0.98<br>(0.49)	1.28<br>(0.18)	0.88<br>(0.62)
Bacterial community composition	na	**3.43**<br>**(<0.01)**	**41.89**<br>**(<0.01)**	0.85<br>(0.60)	0.50<br>(0.90)	0.63<br>(0.80)	0.77<br>(0.67)	0.76<br>(0.68)
Total richness	1.38<br>(0.28)	**5.78**<br>**(0.03)**	**6.83**<br>**(0.02)**	3.16<br>(0.09)	0.42<br>(0.53)	0.01<br>(0.94)	0.01<br>(0.98)	**4.32**<br>**(0.05)**
Fungal richness	2.42<br>(0.07)	3.61<br>(0.08)	**6.14**<br>**(0.02)**	3.16<br>(0.09)	2.88<br>(0.11)	0.84<br>(0.37)	1.21<br>(0.29)	**4.31**<br>**(0.05)**
Bacterial richness	**3.95**<br>**(0.01)**	4.07<br>(0.06)	1.67<br>(0.21)	0.46<br>(0.51)	4.10<br>(0.06)	3.25<br>(0.09)	**5.37**<br>**(0.03)**	0.64<br>(0.43)
Fungal:bacterial	**2.74**<br>**(0.05)**	0.05<br>(0.83)	0.69<br>(0.42)	2.10<br>(0.17)	1.76<br>(0.20)	3.67<br>(0.07)	**5.04**<br>**(0.04)**	1.82<br>(0.20)
Fungal abundance	**4.73**<br>**(<0.01)**	2.68<br>(0.12)	0.36<br>(0.55)	0.16<br>(0.70)	1.33<br>(0.26)	1.24<br>(0.28)	2.16<br>(0.16)	0.19<br>(0.67)

Bacterial abundance	**3.78**	**9.22**	**9.66**	**17.34**	3.45	**10.24**	**11.75**	**18.17**
	**(0.01)**	**(0.01)**	**(0.01)**	**(<0.01)**	(0.08)	**(0.01)**	**(<0.01)**	**(<0.01)**
Xylosidase	**3.47**	**4.39**	**4.52**	**9.03**	**13.50**	**13.92**	**13.94**	**10.22**
	**(0.02)**	**(0.05)**	**(0.05)**	**(0.01)**	**(<0.01)**	**(<0.01)**	**(<0.01)**	**(0.01)**
Sulfatase	**2.84**	3.62	0.80	2.33	0.47	0.002	0.43	0.82
	**(0.04)**	(0.08)	(0.39)	(0.15)	(0.50)	(0.96)	(0.52)	(0.38)
Cellobiohydrolase	0.84	0.87	1.44	0.50	2.68	0.60	1.05	1.05
	(0.57)	(0.37)	(0.25)	(0.49)	(0.12)	(0.45)	(0.32)	(0.32)
ß-glucosidase	**5.04**	0.19	0.71	0.59	**5.10**	3.29	3.93	1.33
	**(<0.01)**	(0.67)	(0.41)	(0.46)	**(0.04)**	(0.09)	(0.06)	(0.27)
∝-glucosidase	2.11	4.11	2.20	2.17	0.50	2.48	1.30	1.99
	(0.10)	(0.06)	(0.16)	(0.16)	(0.49)	(0.13)	(0.27)	(0.18)
Nagase	**4.72**	**7.67**	3.22	**5.55**	0.69	1.07	0.51	**5.42**
	**(<0.01)**	**(0.01)**	(0.09)	**(0.03)**	(0.42)	(0.32)	(0.49)	**(0.03)**
Phosphatase	1.24	0.70	1.55	0.03	3.33	4.02	3.79	0.15
	(0.34)	(0.42)	(0.23)	(0.86)	(0.09)	(0.06)	(0.07)	(0.70)
Phenol oxidase	**3.43**	0.74	1.95	1.07	1.27	1.78	0.89	0.31
	**(0.02)**	(0.40)	(0.18)	(0.32)	(0.28)	(0.20)	(0.36)	(0.59)
Peroxidase	2.23	0.56	1.45	0.001	1.45	0.001	0.001	0.001
	(0.09)	(0.46)	(0.25)	(0.98)	(0.25)	(0.98)	(0.98)	(0.98)
Decomposition rate	**4.06**	2.58	0.26	0.06	0.03	0.005	0.001	0.03
	**(0.01)**	(0.13)	(0.62)	(0.81)	(0.86)	(0.95)	(0.98)	(0.86)

Appendix 2. Warming altered microbial community structure and function at the southern site. Raw data values for all response variables are listed below.

	Southern site									
**Chamber temperature**	**0**	**1.5**	**2.0**	**2.5**	**3.0**	**3.5**	**4.0**	**4.5**	**5.0**	**5.5**
Soil temperature (ºC)	14.1	14.6	14.4	14.9	15.7	14.4	15.6	15.4	15.7	16.1
Soil moisture (VWC)	0.29	0.27	0.26	0.32	0.19	0.17	0.26	0.27	0.23	0.29
Total microbial richness (OTUs)	92	92	98	92	155	79	98	92	83	117
Fungal richness (OTUs)	36	33	43	32	106	24	40	36	28	56
Bacterial richness (OTUs)	56	59	55	60	49	55	58	56	55	61
Fungal:bacterial	2.5	3.2	3.5	6.3	0.2	5.8	1.7	9.0	3.8	1.9
Fungal abundance (copy number g^{-1}soil)	1.89×10^{8}	2.81×10^{8}	1.95×10^{8}	2.75×10^{8}	9.92×10^{7}	1.86×10^{8}	2.25×10^{8}	3.12×10^{8}	1.80×10^{8}	1.05×10^{8}
Bacterial abundance (copy number g^{-1}soil)	1.90×10^{8}	8.78×10^{7}	5.52×10^{7}	4.40×10^{7}	5.53×10^{8}	3.21×10^{7}	1.31×10^{8}	3.47×10^{7}	4.72×10^{7}	5.70×10^{7}
Xylosidase (nmol h^{-1} g $soil^{-1}$)	88.1	189.3	133.8	66.6	16.4	204.2	75.8	183.3	58.3	112.6
Sulfatase (nmol h^{-1} g $soil^{-1}$)	9.6	9.2	14.3	9.2	10.9	11.0	9.8	3.6	0	16.6

Cellobiohydrolase (nmol h^{-1} g $soil^{-1}$)	52.9	124.8	202.9	24.0	5.4	47.5	21.2	194.0	24.2	32.0
ß-glucosidase (nmol h^{-1} g $soil^{-1}$)	93.8	165.4	175.5	77.3	23.7	141.0	104.8	405.5	55.4	80.2
∝-glucosidase (nmol h^{-1} g $soil^{-1}$)	11.0	34.0	24.3	5.2	5.2	4.5	14.4	24.2	3.2	6.7
Nagase (nmol h^{-1} g $soil^{-1}$)	141.5	134.4	97.3	125.7	291.3	200.8	315.3	140.9	53.1	75.2
Phosphatase (nmol h^{-1} g $soil^{-1}$)	671.4	1376.5	1871.7	806.4	1185.9	1372.1	1335.5	1665.2	544.8	900.7
Phenol oxidase (nmol h^{-1} g $soil^{-1}$)	877.8	1343.4	409.6	987.0	738.7	779.9	891.8	2548.2	1834.8	2535.6
Peroxidase (nmol h^{-1} g $soil^{-1}$)	0	0	0	0	0	0	0	0	0	0
Decomposition (k constant)	0.27	0.12	0.36	0.23	0.17	0.35	0.37	0.55	0.13	0.18

Appendix 3. Warming had minimal effect on microbial community structure and function at the northern site. Raw data values for all response variables at the northern site are listed below.

**Chamber temperature**	**Northern site**									
	0	1.5	2.0	2.5	3.0	3.5	4.0	4.5	5.0	5.5
Soil temperature (ºC)	9.5	9.5	10.2	10.5	11.1	10.9	10.3	10.4	11.6	11.1
Soil moisture (VWC)	0.18	0.18	0.14	0.24	0.24	0.14	0.18	0.24	0.17	0.16
Total richness (OTUs)	94	91	133	121	108	105	109	73	92	77
Fungal richness (OTUs)	23	29	53	37	35	34	45	23	17	16
Bacterial richness (OTUs)	70	62	80	84	73	71	64	50	75	61
Fungal:bacterial	8.4	13.2	22.3	4.4	5.6	5.1	5.2	9.6	14.0	3.2
Fungal abundance (copy number g^{-1} soil)	6.25×10^{8}	1.25×10^{9}	1.57×10^{9}	5.89×10^{8}	7.71×10^{8}	4.44×10^{8}	3.65×10^{8}	4.33×10^{8}	1.02×10^{9}	2.84×10^{8}
Bacterial abundance (copy number g^{-1} soil)	7.25×10^{7}	9.47×10^{7}	7.05×10^{7}	1.35×10^{8}	1.38×10^{8}	8.72×10^{7}	7.04×10^{7}	4.50×10^{7}	7.24×10^{7}	8.95×10^{7}
Xylosidase (nmol h^{-1} g soil $^{-1}$)	57.7	41.5	121.9	181.6	123.2	190.1	112.1	77.3	44.5	63.9
Sulfatase (nmol h^{-1} g soil $^{-1}$)	1.1	11.6	6.2	0	0	22.8	0	0.4	0	9.3
Cellobiohydrolase (nmol h^{-1} g soil $^{-1}$)	44.1	47.7	129.5	96.9	83.0	77.9	84.5	20.1	30.9	24.1

ß-glucosidase (nmol h^{-1} g soil $^{-1}$)	253.3	311.9	635.5	396.4	625.4	338.8	403.0	323.5	416.0	143.6
∝-glucosidase (nmol h^{-1} g soil $^{-1}$)	3.2	22.2	2.1	4.9	0	29.4	3.7	0	1.8	2.4
Nagase (nmol h^{-1} g soil $^{-1}$)	396.2	1025.4	698.6	492.7	1646.0	151.7	633.7	422.5	775.4	155.1
Phosphatase (nmol h^{-1} g soil $^{-1}$)	842.1	291.3	889.3	527.6	1299.2	1196.0	966.7	1702.2	1542.3	352.4
Phenol oxidase (nmol h^{-1} g soil $^{-1}$)	195.9	0	0	2768.0	3098.9	0	1248.2	0	0	1073.1
Peroxidase (nmol h^{-1} g soil $^{-1}$)	620.4	0	0	0	0	0	0	0	0	0
Decomposition (k constant)	0.09	0.06	0.14	0.06	0.06	0.04	0.05	0.05	0.09	0.03
